# Overexpression of a carrot BCH gene, *DcBCH1*, improves tolerance to drought in *Arabidopsis thaliana*

**DOI:** 10.1186/s12870-021-03236-7

**Published:** 2021-10-18

**Authors:** Tong Li, Jie-Xia Liu, Yuan-Jie Deng, Zhi-Sheng Xu, Ai-Sheng Xiong

**Affiliations:** State Key Laboratory of Crop Genetics and Germplasm Enhancement, Ministry of Agriculture and Rural Affairs Key Laboratory of Biology and Germplasm Enhancement of Horticultural Crops in East China, College of Horticulture, Nanjing Agricultural University, 1 Weigang, Nanjing, 210095 China

**Keywords:** Carotenoids, β-Carotene hydroxylase, Drought stress, ROS, Abscisic acid synthesis, Carrot

## Abstract

**Background:**

Carrot (*Daucus carota* L.), an important root vegetable, is very popular among consumers as its taproot is rich in various nutrients. Abiotic stresses, such as drought, salt, and low temperature, are the main factors that restrict the growth and development of carrots. Non-heme carotene hydroxylase (BCH) is a key regulatory enzyme in the β-branch of the carotenoid biosynthesis pathway, upstream of the abscisic acid (ABA) synthesis pathway.

**Results:**

In this study, we characterized a carrot BCH encoding gene, *DcBCH1*. The expression of *DcBCH1* was induced by drought treatment. The overexpression of *DcBCH1* in *Arabidopsis thaliana* resulted in enhanced tolerance to drought, as demonstrated by higher antioxidant capacity and lower malondialdehyde content after drought treatment. Under drought stress, the endogenous ABA level in transgenic *A. thaliana* was higher than that in wild-type (WT) plants. Additionally, the contents of lutein and β-carotene in transgenic *A. thaliana* were lower than those in WT, whereas the expression levels of most endogenous carotenogenic genes were significantly increased after drought treatment.

**Conclusions:**

*DcBCH1* can increase the antioxidant capacity and promote endogenous ABA levels of plants by regulating the synthesis rate of carotenoids, thereby regulating the drought resistance of plants. These results will help to provide potential candidate genes for plant drought tolerance breeding.

**Supplementary Information:**

The online version contains supplementary material available at 10.1186/s12870-021-03236-7.

## Background

Carotenoids, mainly including carotenes and xanthophylls, are pigments that are widely found in plants, fungi, and bacteria in nature [1]. In plants, carotenoids are synthesized in plastids. First, pyruvic acid and 3-phosphoglyceraldehyde are used as raw materials to generate the precursor substances for carotenoid synthesis, geranylgeranyl diphosphate (GGPP), through the non-mevalonate (MEP) pathway [2]. Two molecules of GGPP undergo condensation reaction under the action of phytoene synthase (PSY) to produce 15-*cis*-phytoene [3]. 15-*cis*-Phytoene is generated by a series of dehydrogenases and isomerases into all-trans lycopene. Then, all-trans lycopene is further cycled to α-carotene and β-carotene through the action of cyclases, lycopene β-cyclase (LCYB), and lycopene ε-cyclase (LCYE) [4]. Subsequently, the β-ring and ε-ring of α-carotene are catalyzed by carotene hydroxylase to produce α-carotene-derived xanthophylls (lutein). Simultaneously, in the presence of carotene hydroxylase and epoxidase, β-carotene is converted into β-carotene-derived xanthophylls including zeaxanthin, violaxanthin, and neoxanthin [5]. Zeaxanthin is catalyzed by zeaxanthin epoxidase (ZEP) to antheraxanthin, which produces violaxanthin. The violaxanthin can be reconverted to zeaxanthin under violaxanthin de-epoxidase (VDE) catalysis. This process is called the xanthophyll cycle [6, 7]. Violaxanthin and neoxanthin can produce plant hormone, abscisic acid (ABA), under the action of 9-*cis*-epoxycarotenoid dioxygenase (NCED) [8]. Non-heme carotene hydroxylase, BCH (also called CHY, HYD, or HYb), is one type of carotene hydroxylase that is involved in regulating the synthesis of carotenoids in some species. Transgenic tomato fruit hosting the tomato *CrtR-b2* (carotene beta hydroxylase) contained elevated xanthophyll contents [9]. In sweet orange, silencing the expression of β-carotene hydroxylase gene (*Csβ-CHX*) by RNA interference increased the β-carotene content in the pulp of the silenced plant by 36 fold [10].

In nature, widespread abiotic stresses, including drought, salt, high temperature, and low temperature, are factors that severely restrict the normal growth and development of plants. Under stress, reactive oxygen species (ROS) are produced in plants to activate the plant’s defense response. However, when ROS cannot be removed in time and accumulate excessively, they will cause peroxidative damage to the membranes and oxidative damage to other cellular components [11]. In addition, plant endogenous ABA level would increase to trigger the closure of stomata and accumulation of ROS under drought or salt stress [12]. Carotenoids can effectively remove ROS, participate in plant photosynthesis, and provide substrates for ABA synthesis [13, 14]. An association between carotenoids and stress tolerance has also been reported in plants [15]. The homologous overexpression of BCH gene in *Arabidopsis thaliana* increased the content of the xanthophyll cycle pool and enhanced the plant’s tolerance to high temperature and high light [13]. In rice, a T-DNA mutant with β-carotene hydroxylase function loss, *dsm2*, presented drought-hypersensitive phenotype, and the overexpression of *DSM2* significantly improved the drought resistance of rice by promoting the xanthophyll cycle and ABA synthesis [16]. In mulberry, the overexpression of *BCH1* caused a significant increase in the contents of carotenoids and chlorophyll under different stresses, thereby improving plant resistance to different stresses [17].

Carrot (*Daucus carota* L.), one of the top ten vegetable crops in the world, is a rich source of natural antioxidants, including carotenoids, anthocyanins, vitamins, etc. [18]. Carrots are favored by consumers due to their high nutritional and medicinal value [19, 20]. As the demand for carrots increases, improving the yield and quality of carrots is an urgent problem that needs to be solved in carrot production. Drought is one of the key factors restricting the growth and quality of carrots [21]. In the present study, we isolated and characterized a BCH encoding gene, *DcBCH1*, from carrot. Our results showed that the expression level of *DcBCH1* was sharply induced by drought treatment. Transgenic *A. thaliana* plants overexpressing *DcBCH1* possessed higher antioxidant capacity and endogenous ABA content under drought stress. In addition, after drought treatment, the contents of lutein and β-carotene in transgenic plants were lower, whereas the transcriptional levels of most carotene biosynthesis-related genes in transgenic plants were higher compared with those of wild-type (WT) plants. These results indicated that *DcBCH1* can regulate the plants’ tolerance to drought by controlling the synthesis of carotenoids.

## Results

### Isolation and sequence analysis of DcBCH1

**Fig. 1:**
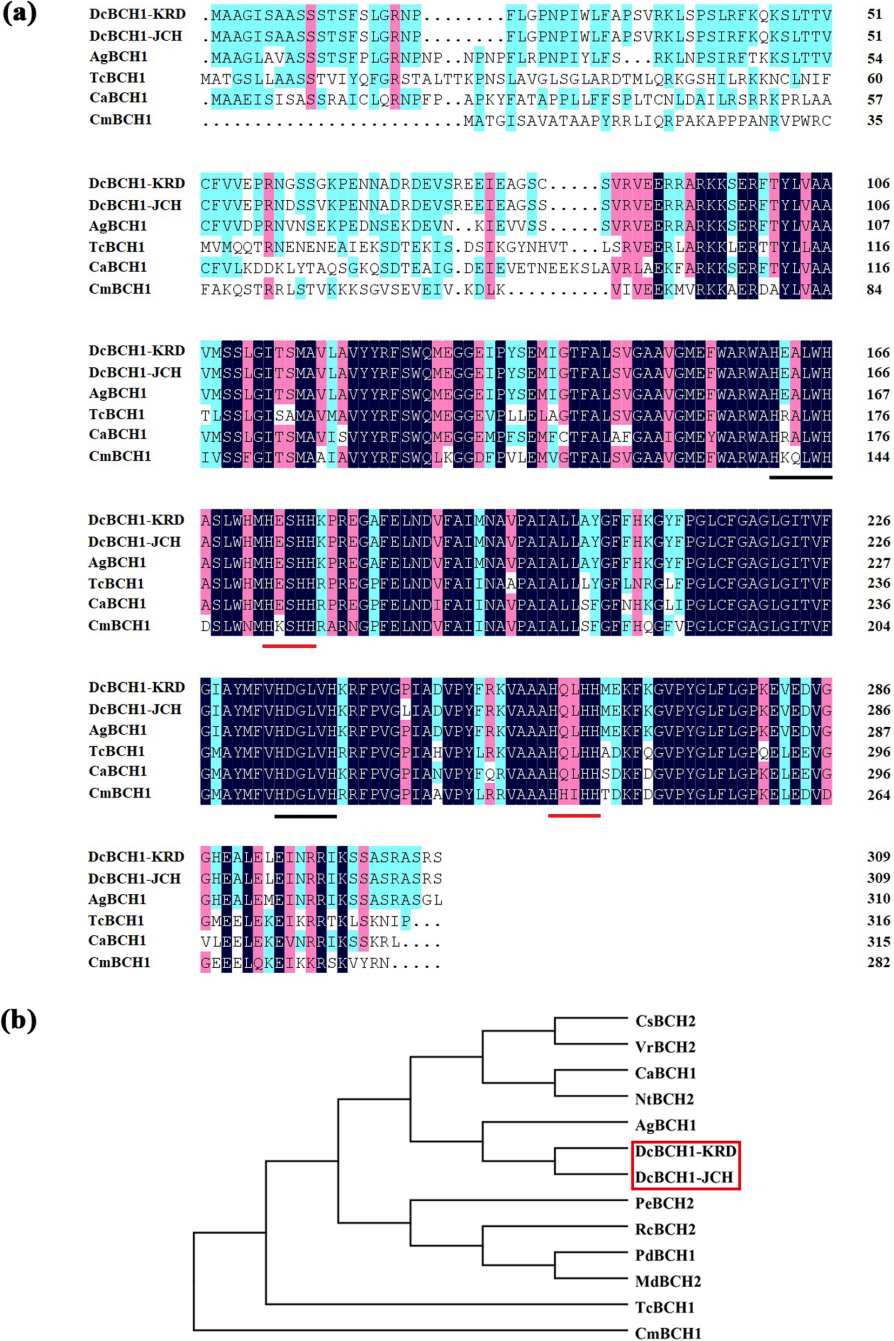
Sequence alignment and phylogenetic relationship between DcBCH1 and other BCHs from various plant species. **a** Multiple sequence alignment of the deduced amino acid sequences of DcBCH1 from carrot (‘Kurodagosun’ (KRD) and ‘Junchuanhong’ (JCH)) with other BCHs from *Prunus dulcis* (PdBCH1, BBG98323.1), *Apium graveolens* (AgBCH1, QDC33551.1), *Theobroma cacao* (TcBCH1, XP_007040333.2), *Capsicum annuum* (CaBCH1, NP_001311784.1), and *Cucurbita moschata* (CmBCH1, XP_022929023.1). The conserved histidine domains ‘HXXXXH’ and ‘HXXHH’ are indicated by black line and red line, respectively. **b** Phylogenetic relationship of the deduced amino acid sequences of DcBCH1from carrot (‘Kurodagosun’ (KRD) and ‘Junchuanhong’ (JCH)) with other BCHs from *Vitis riparia* (VrBCH2, XP_034710058.1), *Camellia sinensis* (CsBCH2, XP_028112187.1), *Nicotiana tabacum* (NtBCH2, XP_016467042.1), *Capsicum annuum* (CaBCH1, NP_001311784.1), *Apium graveolens* (AgBCH1, QDC33551.1), *Prunus dulcis* (PdBCH1, BBG98323.1), *Malus domestica* (MdBCH2, XP_008343769.2), *Rosa chinensis* (RcBCH2, XP_024191328.1), *Populus euphratica* (PeBCH2, XP_011035341.1), *Theobroma cacao* (TcBCH1, XP_007040333.2), and *Cucurbita moschata* (CmBCH1, XP_022929023.1)

The full-length open reading frame (ORF) of *DcBCH1* obtained from carrot (‘Kurodagosun’ and ‘Junchuanhong’) was 930 bp, encoding 309 amino acids, and some differences were observed between the two sequences at the nucleotide and amino acid levels (Additional file 1: Figs. S1–2). Sequence alignment results showed that DcBCH1 from carrot (‘Kurodagosun’ and ‘Junchuanhong’) had the highest similarity with β-carotene hydroxylase from *Apium graveolens* (AgBCH1) and the lowest similarity from *Cucurbita moschata* (CmBCH1). DcBCH1 from carrot (‘Kurodagosun’ and ‘Junchuanhong’) and other five β-carotene hydroxylases from different species all contained two “HXXXXH” (“HEALWH” and “HDGLVH”) and two “HXXHH” (“HESHH” and “HQLHH”) highly conserved histidine domains, which ensure that hydroxylase has catalytic activity (Fig. 1a). Further analysis of the evolutionary relationship of β-carotene hydroxylases in different species showed that DcBCH1 had the closest evolutionary relationship with AgBCH1 (Fig. 1b).

### Expression profiles of *DcBCH1* in carrot

**Fig. 2:**
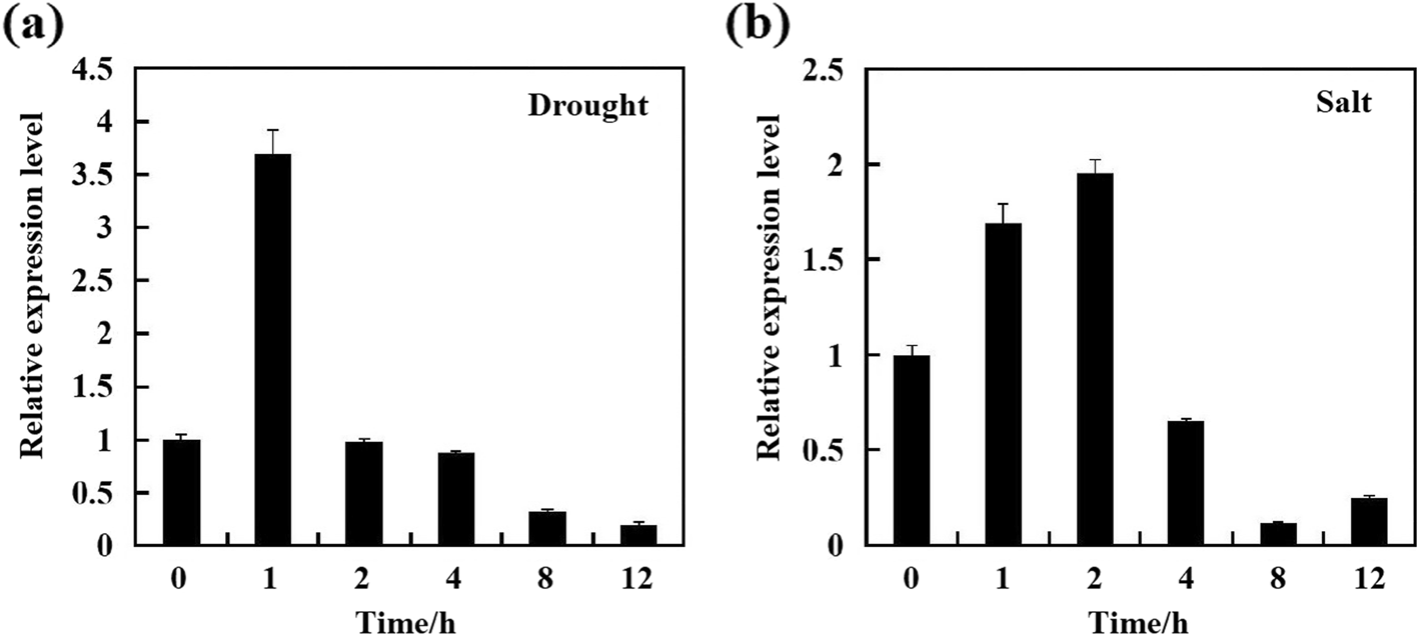
Expression profile of *DcBCH1* under different stress conditions. **a**-**b** The relative expression level of *DcBCH1* under drought (20% PEG) and salt (200 mM NaCl) stress, respectively

RT-qPCR analysis showed that *DcBCH1* expression level was higher in ‘Kurodagosun’ taproot than in ‘Junchuanhong’ taproot (Additional file 1: Fig. S3). Under drought treatment, the transcript level of *DcBCH1* was rapidly induced (3.8-fold to the initial level) 1 h after initiation of the treatment and then decreased gradually (Fig. 2a). Salt treatment also led to a slight increase in *DcBCH1* transcript levels with 2 h (Fig. 2b).

### Generation of *A. thaliana* plants overexpressing *DcBCH1*

**Fig. 3:**
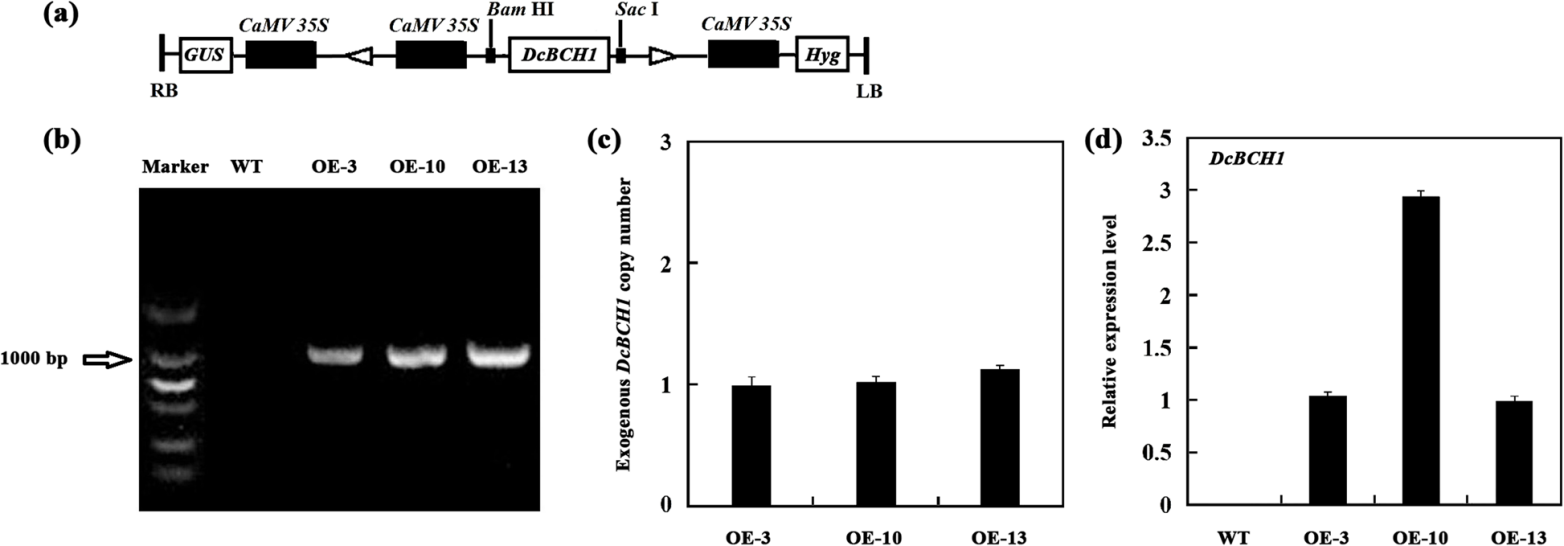
Generation of transgenic *A. thaliana* plants overexpressing *DcBCH1.* **a** Schematic diagram of the overexpression construct. LB, left border; RB, right border. **b** PCR amplification of *DcBCH1* from the cDNA of non-transgenic plants (WT) and transgenic plants (OE-3, OE-10, OE-13). The gel image had high-contrast and the original gel image included in the Additional file 2. **c**-**d** Copy number (**c**) and Relative expression level (**d**) of *DcBCH1* in three independent transgenic *A. thaliana* lines (OE-3, OE-10, OE-13), respectively. Bars represent mean standard deviation (SD)

Given that the expression level of *DcBCH1* was affected by drought treatment, transgenic *A. thaliana* lines overexpressing *DcBCH1* under the control of the CaMV 35S promoter were generated to study the effect of *DcBCH1* upregulation on drought resistance of plants (Fig. 3a). After PCR amplification (Fig. 3b), three independent lines with an overexpressing construct for *DcBCH1* were selected for further analysis. RT-qPCR was used to analyze the copy number and relative expression level of *DcBCH1* in transgenic *A. thaliana* lines. The results showed that the copy number of *DcBCH1* was about one in each of the three transgenic lines, and the transcript level of *DcBCH1* was the highest in OE-10 (Fig. 3c-d).

### *A. thaliana* plants hosting *DcBCH1* present an increased tolerance to drought

**Fig. 4:**
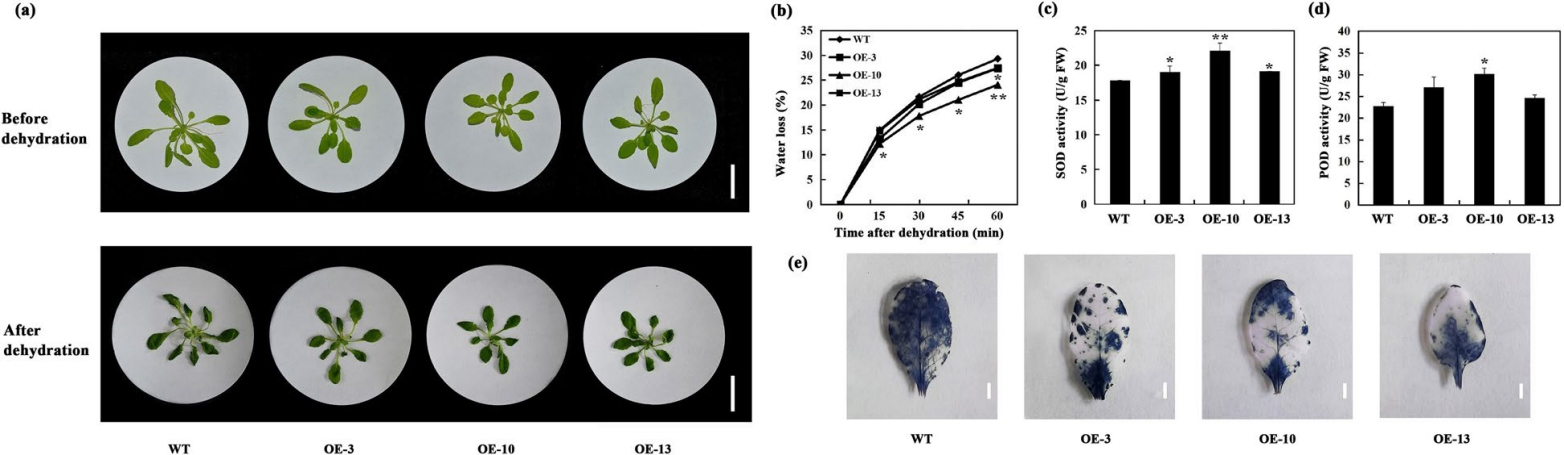
Improved dehydration resistance of *DcBCH1*-overexpression transgenic *A. thaliana* plants. **a** The phenotype from four-week-old WT and transgenic *A. thaliana* plants (OE-3, OE-10, OE-13) before and after 60 min of dehydration. Scale bars = 3 cm. **b** Water loss assays for the whole plants of the WT and transgenic *A. thaliana* plants were performed within 60 min. **c**-**d** The activity of SOD (**c**) and POD (**d**) in the WT and transgenic *A. thaliana* plants measured after dehydration for 60 min. **e** NBT staining for detection the accumulation of O_2_^−^ in WT and transgenic *A. thaliana* (OE-3, OE-10, OE-13) leaves after 60 min of dehydration. Scale bars = 0.5 cm. Bars represent mean standard deviation (SD). Asterisks (*) indicate significant differences between transgenic plants (OE-3, OE-10, OE-13) and the WT plants (* *P* < 0.05; ** *P* < 0.01; *** *P* < 0.001)

After dehydration at room temperature, transgenic and WT plants showed a wilting phenotype, but transgenic plants had lesser degree of wilting (Fig. 4a). Although the fresh weight (FW) of whole transgenic and WT plants decreased, the water loss rate of transgenic *A. thaliana* plants was lower compared with that of WT plants (Fig. 4b). The activity of two key ROS-scavenging enzymes, superoxide dismutase (SOD) and peroxidase (POD), was further measured in transgenic *A. thaliana* and WT plants after dehydration. The results showed that transgenic *A. thaliana* plants had higher enzyme activities of SOD and POD than WT plants (Fig. 4c-d). Consistent with the enzyme activities, damage to the leaf cells of transgenic plants after dehydration was lower than that of WT plants, as evidenced by the greater nitrotetrazolium blue chloride (NBT) staining on WT leaves than on transgenic *A. thaliana* leaves (Fig. 4e).

**Fig. 5:**
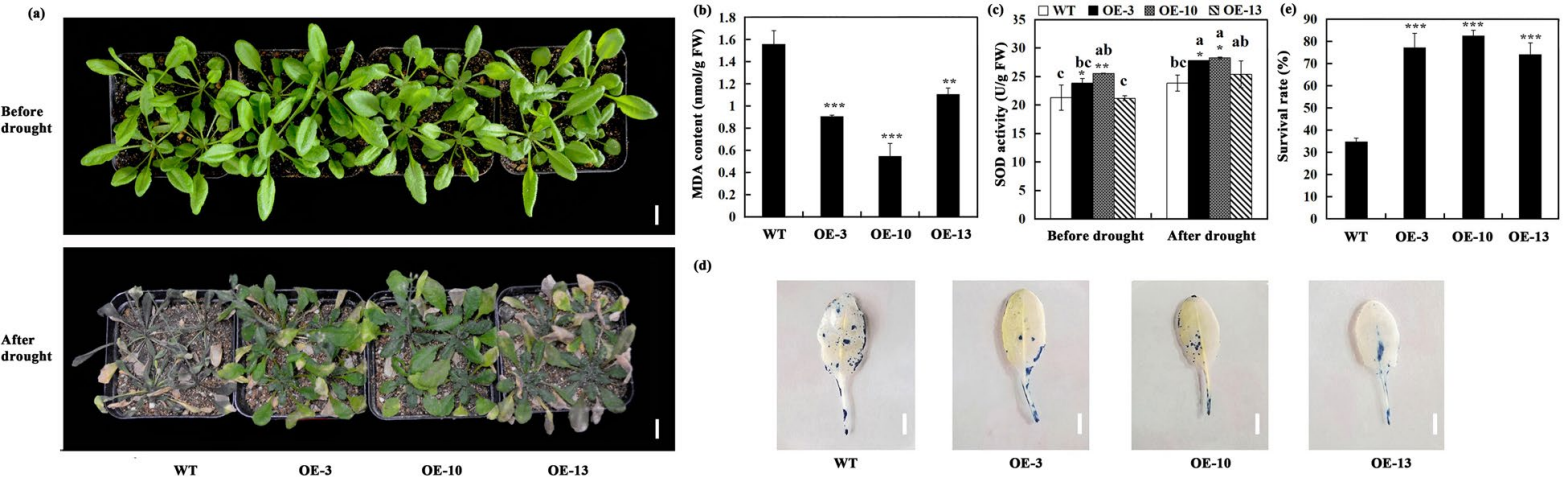
Improved drought resistance of *DcBCH1*-overexpression transgenic *A. thaliana* plants. **a** Appearance of the WT and transgenic *A. thaliana* before and after 15 d of drought treatment. Scale bars = 2 cm. **b** MDA content of WT and transgenic *A. thaliana* (OE-3, OE-10, OE-13) after 15 d of drought treatment. **c** SOD activity of WT and transgenic *A. thaliana* (OE-3, OE-10, OE-13) before and after 15 d of drought treatment. **d** NBT staining for detection the accumulation of O_2_^−^ in WT and transgenic *A. thaliana* (OE-3, OE-10, OE-13) leaves after 15d of drought treatment. Scale bars = 0.5 cm. **e** Survival rate of WT and transgenic *A. thaliana* (OE-3, OE-10, OE-13) after continuous drought and rewatering. Bars represent mean standard deviation (SD). Asterisks (*) indicate significant differences between transgenic plants (OE-3, OE-10, OE-13) and the WT plants (* *P* < 0.05; ** *P* < 0.01; *** *P* < 0.001). The letters above the bars indicate significant differences between the plant lines (*P* < 0.05, according to Tukey’s multiple range test)

Similar results were observed in the experiment of natural drought stress treatment. WT plants showed severe wilting phenotype, while transgenic *A. thaliana* plants showed mild drought stress symptoms (slightly wilting and some leaves were yellow) after depriving water for 15 days (Fig. 5a). On the basis of the above results, we checked the degree of superoxide anion radical (O_2_^−^) accumulation in the leaves of transgenic *A. thaliana* and WT plants, and observed that the accumulation of O_2_^−^ in WT plants was higher than that in transgenic *A. thaliana* plants. The number of blue spots on the leaves of WT plants was greater than that of transgenic *A. thaliana* plants (Fig. 5d). The content of malondialdehyde (MDA) was also lower in transgenic *A. thaliana* plants than in WT plants (Fig. 5b). Moreover, under normal and drought conditions, the SOD activity of transgenic *A. thaliana* plants was higher than that of WT plants (Fig. 5c). We also recorded higher survival rate in transgenic *A. thaliana* plants than in WT plants after drought treatment and rewatering (Fig. 5e).

### Effect of drought stress on endogenous ABA level and lutein and β-carotene contents

**Fig. 6:**
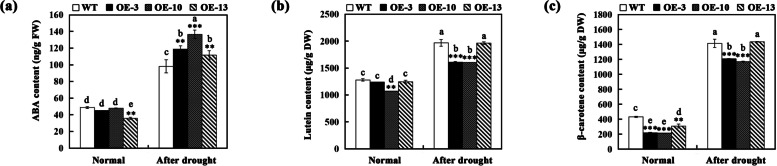
Endogenous ABA level and carotenoids (lutein, β-carotene) content in WT and transgenic *A. thaliana* plants under normal condition and drought stress. **a** Endogenous ABA level in WT and transgenic *A. thaliana* (OE-3, OE-10, OE-13) plants under normal condition and after 15 d of drought treatment. **b**-**c** Lutein (**b**) and β-carotene (**c**) content in WT and transgenic *A. thaliana* (OE-3, OE-10, OE-13) plants under normal condition and after 15 d of drought treatment. Bars represent mean standard deviation (SD). Asterisks (*) indicate significant differences between transgenic plants (OE-3, OE-10, OE-13) and the WT plants (* *P* < 0.05; ** *P* < 0.01; *** *P* < 0.001). The letters above the bars indicate significant differences between the plant lines (*P* < 0.05, according to Tukey’s multiple range test)

The ABA content of WT and transgenic *A. thaliana* plants under normal condition and after drought treatment was measured. As shown in Fig. 6a, under normal condition, ABA contents in transgenic *A. thaliana* plants were similar to those in WT plants (OE-13 lower than WT). After drought treatment, transgenic *A. thaliana* plants accumulated about 111–136 ng g^− 1^ FW of ABA, while WT plants accumulated less ABA content (98 ng g^− 1^ FW). The accumulation of lutein and β-carotene in WT and transgenic *A. thaliana* plants under normal condition and after drought treatment was also determined. The results showed that the contents of lutein and β-carotene in transgenic *A. thaliana* plants decreased to varying degrees compared with those in WT plants (Fig. 6b-c).

### Effect of drought stress on endogenous carotenogenic gene expression in transgenic *A. thaliana* and WT plants

**Fig. 7:**
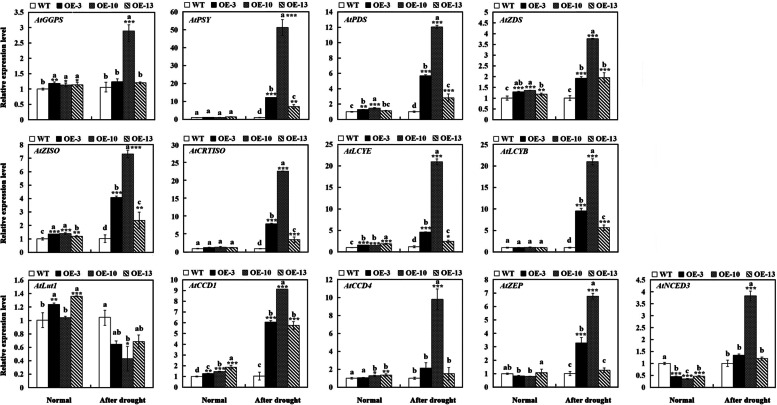
Expression patterns of carotenogenic genes in WT and transgenic *A. thaliana* plants under normal condition and drought stress. Transcript levels of endogenous carotenogenic genes in WT and transgenic *A. thaliana* (OE-3, OE-10, OE-13) under normal condition and after 15 d of drought treatment. Bars represent mean standard deviation (SD). Asterisks (*) indicate significant differences between transgenic plants (OE-3, OE-10, OE-13) and the WT plants (* *P* < 0.05; ** *P* < 0.01; *** *P* < 0.001). The letters above the bars indicate significant differences between the plant lines at each time point (*P* < 0.05, according to Tukey’s multiple range test)

Moreover, we investigated the transcript levels of 13 carotenogenic genes in WT and transgenic plants. Under normal condition, the mRNA levels of *AtPDS*, *AtZDS*, *AtZISO*, and *AtCRTISO* in transgenic plants were significantly higher than those in WT plants. After drought treatment, many genes positioned upstream or downstream of the β-carotene hydroxylation step, such as *AtPSY*, *AtPDS*, *AtZDS*, *AtZISO*, *AtCRTISO*, *AtLCYE*, *AtLCYB*, *AtZEP*, *AtCCD1*, and *AtCCD4*, were significantly higher in transgenic *A. thaliana* plants, whereas the transcript level of *AtLut1* was lower compared with those in WT plants. *AtNCED3* expression level in transgenic *A. thaliana* plants exhibited opposite results compared with WT plants under normal condition (decreased to 35–50% in transgenic plants) and after drought treatment (increased 1.2–3.8 fold in transgenic plants) (Fig. 7).

## Discussion

Carrot is one of the most important vegetables due to its various nutrients. In the process of carrot planting, drought is a key limiting factor for its yield and quality. For example, under drought stress, the degree of lignification of carrot fleshy roots will increase, resulting in a decrease in its quality [22]. During the long evolution of plants, a series of complex regulatory mechanisms has been produced in plants to adapt to the continuous changes in the environment [23]. When plants encounter stress, their physiological and biochemical states will change to different degrees to improve their survival rate under stress [24]. Carotenoids have powerful antioxidant functions due to their polyunsaturated conjugated double bond molecular structure and play an important role in the response of plants to stress [25]. The accumulation of carotenoids is affected by changes in the transcription levels of key enzymes involved in the carotenoid biosynthesis pathway [26, 27]. Omics analysis is an effective method for identifying genes related to quality or stress resistance [28, 29]. BCH, includes BCH1 and BCH2, has been identified in some species, and it can cause changes in carotenoid accumulation and participate in the process of plant response to stress [16, 30, 31].

In the present study, we isolated the BCH1 encoding gene from carrot and named it *DcBCH1*. Research identified that the amino acid sequence of BCH with normal catalytic function contains two “HXXXXH” and two “HXXHH” highly conserved histidine domains. The deletion or change of amino acid residues will cause the enzyme to lose its catalytic activity [32]. Multiple alignment results showed that the DcBCH1 from carrot (‘Kurodagosun’ and ‘Junchuanhong’) has high similarity with other BCHs from different species, and the amino acid sequence contains four complete conserved domains. RT-qPCR results showed that the expression level of *DcBCH1* was induced by drought treatment. These results indicated that *DcBCH1* may be involved in plant response to drought stress.

We further overexpressed *DcBCH1* heterologously in *A. thaliana*. The transgene is randomly inserted into the recipient plant with one or more copies. When the transgene is inserted into the recipient plant with a single copy, its expression level is higher, and the genetic stability is better. However, the insertion of multiple copies of the transgene usually causes the expression of transgene in recipient plants to be weakened or unstable, resulting in gene silencing [33–35]. Our results showed that the copy number of *DcBCH1* in the three transgenic lines was about one, indicating that *DcBCH1* can be stably inherited and expressed in transgenic plants. Oxidative stress caused by excessive accumulation of ROS including hydrogen peroxide (H_2_O_2_), O_2_^−^, and hydroxide anions (OH^−^) can lead to lipid peroxidation under dehydration or drought stresses. MDA is one of the most important products of membrane lipid peroxidation, and its accumulation can be used to judge the degree of membrane lipid peroxidation [36]. Carotenoids can eliminate ROS, thereby protecting plant tissue cells from peroxidation damage [37]. In rice, compared with WT plants, transgenic plants that overexpress a BCH gene (*dsm2*) contained higher xanthophyll content and had higher activity of SOD and lower MDA accumulation, while the *dsm2* mutant plant showed the opposite results under oxidative stress [16]. In this study, the transgenic *A. thaliana* had higher SOD and POD activities and accumulated less O_2_^−^ after drought and dehydration stress. In addition, after drought stress, the peroxidation damage degree of transgenic *A. thaliana* was significantly lower than that of WT plants (evidenced by MDA content). We observed that the SOD activity of the OE-10 line was the highest before and after drought stress. Further analysis of the increase in SOD activity before and after drought stress showed that WT had the lowest SOD activity, followed by OE-10, OE-3, and OE-13.

Plants have two types of defense systems that work together to resist the damage caused by ROS: enzymes and non-enzymes [37, 38]. The antioxidant capacity of carotenoids depends on the number and molecular structure of conjugated double bonds in their molecules, as well as their position in the lipid membrane [39, 40]. Zeaxanthin (β-carotene-derived xanthophyll) has better antioxidant protection ability because its molecular structure can cross the membrane [41]. In this study, results showed that under normal condition and after drought treatment, the β-carotene and lutein in the transgenic lines were lower than those in WT plants. Among the three transgenic lines, OE-10 had the lowest β-carotene content. In *Escherichia coli* BL21 (DE3), CitHYb could catalyze the hydroxylation of the β-rings of β-carotene and α-carotene [31]. *CrtR-b2*-overexpressed tomato leaves contained lower levels of lutein and β-carotene and higher levels of β-carotene-derived xanthophylls (violaxanthin) than control plants [9]. In transgenic orange with silenced *Csβ-CHX* expression level, β-carotene content was significantly increased, while β,β-xanthophylls and ε,β-xanthophylls contents were significantly decreased compared with control plants [10]. These results suggested that the content of β-carotene-derived xanthophylls is increased in our transgenic plants. Transgenic tobacco with *chyB* overexpression contained higher zeaxanthin content, and the increase of zeaxanthin content improved the total antioxidant capacity of plants in the lipid phase and enhanced the tolerance of plants to drought stress [30]. On the basis of the above results, we speculated that the lowest increase of SOD activity in OE-10 may be because the excessive ROS produced under drought conditions are preferentially cleared by β -carotene-derived xanthophyll.

The levels of carotenoids, including total carotenoids, α-carotene, and β-carotene, are affected by the total flux of the pathway and the activity and stability of related enzymes [42]. PSY is the most critical rate-limiting enzyme in carotenoid synthesis, and its activity determines the total amount of carotenoids in plant tissues [43]. The overexpression of a bacterial *PSY* in tomato significantly increased the total carotenoid content in tomato fruit [44]. In transgenic cassava roots, *PSY* overexpression increased the content of carotenoids [45]. The relative content and activity of LCYB and LCYE enzymes largely determine the ratio of β-carotene and α-carotene [46, 47]. The overexpression of *DcLcyb1* in carrot directed the carotenoid flux to the synthesis to β-carotene, resulting in increased β-carotene content in the roots and leaves of transgenic carrots [26]. In *A. thaliana*, *LUT1* encoding CYP97C1 was confirmed to be responsible for the hydroxylation of the ε-ring, and the leaves of *lut1* mutant *A. thaliana* contained lower lutein and higher β-xanthophylls compared with WT plants [48]. Our results showed that under drought condition, the expression level of most carotenoid biosynthesis genes in transgenic plants was higher than that in WT plants, while the expression level of *AtLut1* in transgenic plants was lower than that in WT plants. These results suggested that the flux of biosynthesis of β-carotene-derived xanthophylls was increased in transgenic plants overexpressing *DcBCH1*. In sweetpotato, suppression of the β-carotene hydroxylase gene (*CHY-β*) increased the content of β-carotene, and the expression levels of some upstream genes (*PSY*, *PDS*, *ZDS*) of *CHY-β* in transgenic sweetpotato were significantly lower than those in control plants [49]. In this study, the expression levels of *DcBCH1* upstream genes (*AtPSY*, *AtPDS*, *AtZDS*, *AtZISO*, and *AtCRTISO*) in transgenic plants were higher than those in WT plants after drought treatment. Carotenoid cleavage dioxygenase (CCD) is one of the main enzymes that degrade carotenoids in plants; moreover, it can specifically cleave double bonds on carotenoids to produce a variety of apo-carotenoids [50]. We found that the overexpression of *DcBCH1* increased the transcript levels of *AtCCD1* and *AtCCD4* under drought stress condition. All these results indicated that the biosynthesis and degradation rate in transgenic plants were greater than those in WT plants. The increased resistance of transgenic *A. thaliana* to drought may be caused by the overexpression of *DcBCH1*, which makes the synthesis and metabolism of carotenoids faster, thereby giving the plant stronger antioxidant capacity.

Increasing number of evidence showed that ABA acts as a signal molecule for plants to respond to stress [51, 52]. In our study, the endogenous ABA level in transgenic *A. thaliana* plants (expect OE-13) was similar to that in WT under normal conditions, while it was significantly higher than that in WT plants after drought treatment. *AtNCED3* was found to play a key role in ABA biosynthesis under drought-stressed conditions in *A. thaliana* [53]. Transgenic sweetpotato with higher *NCED* transcription levels also had higher ABA levels [49]. Our results showed that *AtNCED3* transcript level in transgenic *A. thaliana* plants was lower than that in WT under normal condition, while its transcript level in transgenic plants with higher endogenous ABA content was higher than that in WT after drought treatment. Research showed that under normal condition, a basal level of ABA in plants is required to regulate stomata and participate in plant growth and metabolic pathways [54, 55]. A rigorous regulatory system exists in plants to control carotenoid synthesis and maintain a steady state [56]. Under normal condition, the expression level of some upstream genes (*AtPDS*, *AtZDS*, *AtZISO*, and *AtCRTISO*) of *DcBCH1* in transgenic plants was higher than that in WT plants, which may lead to the accelerated rate of carotenoid synthesis in transgenic plants. Moreover, the decreased expression level of *AtNCED3* may play a role in maintaining the ABA level in a steady state. After drought treatment, the expression level of *AtNCED3* in transgenic plants was higher than that in WT, which was consistent with the higher ABA level in transgenic plants than in WT plants after drought treatment. In addition, the expression of *AtZEP* was similar to that of *AtNCED3* in transgenic plants compared with WT. These above results indicated that in response to drought stress, *DcBCH1* overexpression increases the rate of carotenoid synthesis, provides a large amount of precursor substances for ABA synthesis, and promotes ABA accumulation, thereby increasing the plant’s resistance to drought.

In summary, this study reports the function of *DcBCH1* from carrot in regulating the drought resistance of plants. The overexpression of *DcBCH1* in *A. thaliana* improved transgenic plants’ tolerance to drought by regulating the synthesis of carotenoids to control antioxidant capacity. Our results will help to provide important candidate genes for plant drought tolerance breeding.

## Materials and methods

### Plant materials and growth condition

Two carrot cultivars (‘Kurodagosun’ and ‘Junchuanhong’) were selected as materials. Carrot cv. (‘Kurodagosun’ and ‘Junchuanhong’) and *A. thaliana* (Columbia ecotype) were deposited at the State Key Laboratory of Crop Genetics and Germplasm Enhancement, Nanjing Agricultural University (32°04′N, 118°85′E). Seeds of two carrot cultivars were sown into pots and grown in a growth chamber. The climate parameters of the growth chamber are 12 h light (25 °C)/12 h dark (18 °C) with 320 μmol m^− 2^ s^− 1^ light intensity. The taproot of ‘Kurodagosun’ and ‘Junchuanhong’ were sampled at 110 d after sowing. To analyze the transcript levels of *DcBCH1* under stress, plants of ‘Junchuanhong’ at 60-day old were treated with abiotic stresses. For drought and salt stresses, the plants were irrigated with 20% polyethylene glycol (PEG) and 200 mM NaCl solution, respectively, and sampled at 0, 1, 2, 4, 8, and 12 h after treatment. All the samples were quickly frozen in liquid nitrogen and stored at − 80 °C for further analysis.

### Total RNA extraction, gene cloning, and reverse transcription quantitative real-time PCR (RT-qPCR) analysis

The total RNA from samples were extracted using Total RNA extraction kit (Tiangen, Beijing, China), and then converted into cDNAs using Prime Script RT reagent kit (Vazyme, Nanjing, China) according to the manufacturer’s instructions. Combining carrot transcriptome and genomic data [57–59], the sequence of *DcBCH1* was obtained, and a pair of specific primers (Table 1) was designed to clone *DcBCH1* from carrot cultivar (‘Kurodagosun’ and ‘Junchuanhong’) by RT-PCR. The RT-PCR program consists of 3 min at 98 °C, followed by 34 cycles of 10 s at 98 °C, 30 s at 55 °C, 15 s at 72 °C and a 10 min extension at 72 °C. The PCR product was analyzed by agarose gel electrophoresis and subsequently sequenced in genscript (Nanjing, China). RT-qPCR assays were performed according to the method described previously [61]. *DcActin* and *AtActin8* were used as internal control to normalize the expression levels of the target genes in carrot and *A. thaliana*, respectively [62, 63]. Three technical replicates for each experiment were performed in three biological replicates. The primers used for RT-qPCR assays of carotenoid biosynthesis genes in *A. thaliana* were referenced from previous study and listed in Table 1 [60].

**Table 1:** Primer sequences used in this study

Gene	Function	Forward primer (5′-3′)	Reverse primer (5′-3′)	Reference
*DcBCH1*	Full lengths clone	ATGGCGGCCGGAATTTCGGCG	CTATGATCGGCTAGCTCTGGA	–
*DcBCH1*	Overexpression vector conduction	TTTACAATTACCATGGGATCCATGGCGGC CGGAATTTCGGCG	ACCGATGATACGAACGAGCTCCTATGATCGGC TAGCTCTGGA	–
*AtGGPS*	RT-qPCR	TGCTTGTGAACTCGTCGGAGGT	CACGGCGGAGATCGTCGTTATC	–
*AtPSY*	RT-qPCR	TGCTGCTCTCGCTGATACAGTTG	CCTCTTCTCGCATCTTCGCCTAC	Yin et al. 2020 [60]
*AtPDS*	RT-qPCR	GCTCAATGACGATGGCACGGTTA	CGGCATACACGCTCAGAAGGTTAC	Yin et al. 2020 [60]
*AtZDS*	RT-qPCR	GACATTCGCAACCTTGACAGCATAA	CCTTGAGCATACGCAACAGAGAAG	Yin et al. 2020 [60]
*AtZISO*	RT-qPCR	CTAACCTCACCTTAATCCGCCGTAT	AAGAGAACAACTCCAAGGACAACAC	Yin et al. 2020 [60]
*AtCRTISO*	RT-qPCR	TCCTCAGCATTCCAACCATTCTTGA	GCGAGTGTCCTTAGCCAACCAA	Yin et al. 2020 [60]
*AtLCYE*	RT-qPCR	GTAGTGTCAGAGCTAGCGGC	AAGGCTAAACCAGCAGGACC	–
*AtLCYB*	RT-qPCR	AGATGGAATGTGCCTTGTTGTTGGA	ACTCGGAGATGTTGATTGCGGTTC	Yin et al. 2020 [60]
*AtLut1*	RT-qPCR	GCGTCTCTATCCTCATCCTCCT	GTATGGTTGCTCCTGTGGTCAT	–
*AtCCD1*	RT-qPCR	TGGCAGCAGCATCATCTCAGTC	ATCACGGATGGGAGCGAAGTTG	–
*AtCCD4*	RT-qPCR	CGGCACTCTTCCACTGTCACTT	TTCGGTTAATCGGACGGCGTAG	–
*AtZEP*	RT-qPCR	TCTTCGTTGACATTGCTATGCCATC	CGCCGCCTTCTTATCTGAACCA	Yin et al. 2020 [60]
*AtNCED3*	RT-qPCR	GGAGATGGCTTGGTGGCAATCA	GCTTCTCGTGGCTGACAAGGAA	–

### Biological information analysis of DcBCH1

Nucleotide and amino acid sequences of BCHs from other species retrieved from the NCBI database (http://www.ncbi.nlm.nih.gov). Alignment of amino acid sequences of BCH1s was conducted using the DNAMAN 6.0 software. The phylogenetic tree of BCHs from different species was constructed using the neighbor-joining method of MEGA 5.0 [64].

### Generation of transgenic *A. thaliana*

Using a pair of specific primers (Table 1) to PCR amplify the full length of the *DcBCH1* ORF from ‘Kurodagosun’ and insert it into the pCAMBIA1301 vector which containing the 35S cauliflower mosaic virus (CaMV) promoter and a hygromycin (kanamycin) resistance marker to create *35S:DcBCH1* construct. The recombinant vector was introduced into *A. tumefaciens* (GV3101) by the electroporation method. The *35S:DcBCH1* construct was transformed into *A. thaliana* to produce transgenic *A. thaliana* plants that overexpress *DcBCH1* in accordance with a previously described method [65]. Transgenic *A. thaliana* plants were detected by PCR amplification and RT-qPCR analysis. The copy number of *DcBCH1* in each transgenic lines was detected in accordance with a previously described method [66]. The single copy gene, *4-HPPD*, in *A. thaliana* was used as the control gene [67]. Genomic DNA was extracted from the leaves of each transgenic lines, and diluted the DNA into fivefold, 5^2^-fold, 5^3^-fold, 5^4^-fold. The *C*q values of *4-HPPD* and *DcBCH1* were detected by RT-qPCR, and the standard curves of *4-HPPD* and *DcBCH1* were established based on the respective *C*q values of DNA with different dilution gradients and the corresponding logarithm of the dilution multiples. The number of copies was calculated according to the formula: *X*_*0*_*/R*_*0*_ = 10^[(*Cq,X*-*IX*)/*SX*]-[(*Cq,R*-*IR*)/*SR*]^ [68]. The average value of *X*_*0*_*/R*_*0*_ was the copy number of *DcBCH1* in transgenic line*.* Three technical replicates for each experiment were performed. Three transgenic lines overexpressing *DcBCH1* were screened until homozygous seeds were obtained (T_3_ generation) for further analysis.

### Evaluation of transgenic *A. thaliana* tolerance to drought stress

For drought stress testing of transgenic *A. thaliana* at the seedling stage, two experiments were designed. Plants of WT and three transgenic *A. thaliana* lines were planted in the mixed soil (soil, perlite, and vermiculite (16:9:1, v/v/v)) and grown in a growth chamber maintained at 22 °C/18 °C under light conditions (12 h light/12 h dark cycle). When *A. thaliana* grew to four-week old, the seedlings of WT and transgenic *A. thaliana* were removed from the soil, cleaned the soil on the roots, and dried at room temperature for 60 min for dehydration treatment. Measure the FW of the seedlings every 15 min and calculate the relative water loss rate. In addition, the seedlings of WT and transgenic *A. thaliana* planted in the mixed soil were normally watered, when they grew to four-week old, watering was stopped to allow the development of drought stress. After the two drought stress experiments were completed, the phenotype and relevant physiological parameters were observed and measured, respectively. After continuous drought for 15 d and then rewatering, record the survival of WT and transgenic plants and calculate the survival rate. Three biological replicates were used for recording all the observations.

### Physiological measurements and NBT staining assay

The determination and calculation of plant water loss rate, antioxidant enzyme (SOD and POD) activity, and MDA content after dehydration or drought treatment were carried out according to our previously described methods [61]. All leaves of WT and transgenic *A. thaliana* plants before and after dehydration or drought stress treatment were collected for antioxidant enzyme activity and MDA content measurement. NBT staining was used to analyze the accumulation of O_2_^−^ in *A. thaliana* leaves. In brief, after dehydration or drought treatment, the 8th leaf of WT and transgenic *A. thaliana* plants was immersed in NBT staining solution, incubated with shaking at 25 °C in the dark for 16 h. After staining, the leaves were placed in 95% alcohol for decolorization and observation. Three technical and biological replicates were performed for each physiological measurement.

### ABA extraction and measurement

All leaves of WT and transgenic *A. thaliana* plants were sampled under normal condition and after drought stress treatment to measure the endogenous ABA level. The extraction and determination of ABA in samples were performed by using the indirect enzyme-linked immunosorbent assay method as described previously with three technical and biological replicates [69].

### Measurement of carotenoid contents

All leaves from WT and transgenic *A. thaliana* plants were collected for carotenoid extraction in accordance with the method described by Ma et al. [70]. The β-carotene and lutein were separated using a Thermo UltiMate UHPLC System (Thermo, USA) with a Hedera ODS-2 C18 column (250 mm X 4.6 mm, 5 μm nominal particle size; Shimadzu, Japan), operated at a flow rate of 1 mL min^− 1^ at 30 °C with mobile phase containing methanol: acetonitrile (90:10, v/v). Detection was performed at 450 nm. Three biological replicates were used for carotenoids contents measurement.

### Statistical analysis

For analyzing the significant differences in gene expression and physiological parameters between WT and *DcBCH1*-overexpressing *A. thaliana* plants, the one-way ANOVA with Tukey’s post-test was carried out at the significance levels *P* < 0.05 (*), *P* < 0.01 (**), and *P* < 0.001 (***). The plant lines were compared using Tukey’s multiple range test (*P* < 0.05).

## Supplementary Information

**Additional file 1: Fig. S1** Nucleotide acid and deduced amino acid sequence of *DcBCH1* from ‘Kurodagosun’. **Fig. S2** Nucleotide acid and deduced amino acid sequence of *DcBCH1* from ‘Junchuanhong’. **Fig. S3** The expression level of *DcBCH1* in ‘Junchuanhong’ and ‘Kurodagosun’.

**Additional file 2: Fig. S4** The original gel image for PCR amplification of *DcBCH1* from cDNA of non-transgenic (WT) and transgenic plants (OE-3, OE-10, OE-13).

## Data Availability

*DcBCH1* sequence data from ‘Kurodagosun’ in this study has been submitted to the NCBI database with accession no. MW014363. Sequence data used in this article can be found in the GenBank database (http://www.ncbi.nlm.nih.gov/Genbank) under the following accession numbers: PdBCH1 (BBG98323.1); AgBCH1 (QDC33551.1); TcBCH1 (XP_007040333.2); CaBCH1 (NP_001311784.1); CmBCH1 (XP_022929023.1); VrBCH2 (XP_034710058.1); CsBCH2 (XP_028112187.1); NtBCH2 (XP_016467042.1); MdBCH2 (XP_008343769.2); RcBCH2 (XP_024191328.1); PeBCH2 (XP_011035341.1) and TcBCH1 (XP_007040333.2). The data sets supporting the conclusions of this article are included within the article and its additional files. *A. thaliana* (Columbia ecotype) and carrot (‘Kurodagosun’ and ‘Junchuanhong’) were deposited at the State Key Laboratory of Crop Genetics and Germplasm Enhancement, Nanjing Agricultural University.

## Abbreviations

GGPP: Geranylgeranyl diphosphate
MEP: Mevalonate
ABA: Abscisic acid
ROS: Reactive oxygen species
WT: Wild type
ORF: Open reading frame
SOD: Superoxide dismutase
POD: Peroxidase
O_2_^−^: Superoxide anion radicals
MDA: Malondialdehyde
OH^−^: Hydroxide anions
NBT: Nitrotetrazolium blue chloride
H_2_O_2_: Hydrogen peroxide
FW: Fresh weight
