# Supplementary material for: Overexpression of a carrot BCH gene, DcBCH1, improves tolerance to drought in Arabidopsis thaliana
Source: BMC Plant Biol. 2021 Oct 18;21:475. doi: 10.1186/s12870-021-03236-7 (PMC8522057; doi:10.1186/s12870-021-03236-7)
Supplement: Supplementary file 1 — Additional file 1: Fig. S1 Nucleotide acid and deduced amino acid sequence of DcBCH1 from ‘Kurodagosun’. Fig. S2 Nucleotide acid and deduced amino acid sequence of DcBCH1 from ‘Junchuanhong’. Fig. S3 The expression level of DcBCH1 in ‘Junchuanhong’ and ‘Kurodagosun’. [file 12870_2021_3236_MOESM1_ESM.doc]

**Additional file 1:**

At the nucleotide level, the 26 base site differences are, C / A (position 27, 121, 354, 699), G / A (position 34, 117, 267, 456, 537), A / T (position 96), A / G (position 113, 318, 672), T / C (position 123, 759, 300), G / C (position 169, 250), G / T (position 188), C / T (position 490, 737, 741, 744), T / A (position 585, 591), C / G (position 207), respectively. At the amino acid level, 7 site differences are, A / T (position 12), N / S (position 38), L / I (position 41), A / P (position 57), G / V (position 63), C / S (position 84), P / L (position 246).

1 atggcggccggaatttcggcggcctc**c**agctcc**g**cctcattctctctgggccgcaacccatttctcgggcccaaccccatttggctcttc

M A A G I S A A S S S **A** S F S L G R N P F L G P N P I W L F

91 gctcc**a**tccgtccgaaaactaa**a**ccc**g**tct**c**t**t**cggttcaaacaaaagagcttaaccactgtttgttttgtggtggag**g**cccgaaatgat

A P S V R K L **N** P S **L** R F K Q K S L T T V C F V V E **A** R N D

181 agctcgg**g**taagcccgaaaataacgc**c**gaccgagatgaagtgagtcgcgaggagattgaggcggggagtt**g**ttccgtacgagttga**g**gag

S S **G** K P E N N A D R D E V S R E E I E A G S **C** S V R V E E

271 agaagagcgaggaagaagtcggagaggtt**t**acgtatttggtggcggc**a**gttatgtcgagtcttggcattacttctatggctgt**c**ttggcg

R R A R K K S E R F T Y L V A A V M S S L G I T S M A V L A

361 gtttattacagattctcttggcaaatggagggtggcgaaattccgtattcggagatgattggtacattcgctctttctgttggtgctgct

V Y Y R F S W Q M E G G E I P Y S E M I G T F A L S V G A A

451 gtggg**g**atggaattttgggcgagatgggcacatgaagcc**c**tgtggcatgcttcgttgtggcatatgcacgagtcacaccataaacc**g**aga

V G M E F W A R W A H E A L W H A S L W H M H E S H H K P R

541 gaaggagcatttgagctgaatgatgtatttgcaataatgaacgc**t**gttcc**t**gcaatagctttgctagcttatggtttcttccacaaaggc

E G A F E L N D V F A I M N A V P A I A L L A Y G F F H K G

631 tattttccaggtctctgttttggtgcgggcctgggaatcac**a**gtatttggaatcgcttacatgtttgt**c**cacgatggtcttgttcataaa

Y F P G L C F G A G L G I T V F G I A Y M F V H D G L V H K

721 cgatttccggtaggtc**c**cat**c**gc**c**gatgtcccttactt**t**agaaaagttgctgctgctcatcagctgcaccacatggaaaagttcaaagga

R F P V G **P** I A D V P Y F R K V A A A H Q L H H M E K F K G

811 gtcccatatgggttgtttttgggtcctaaggaagttgaggatgtgggaggacacgaagcgttagaattggagatcaacagaagaatcaag

V P Y G L F L G P K E V E D V G G H E A L E L E I N R R I K

901 tcatctgcttccagagctagccgatcatag

S S A S R A S R S *

**Fig. S1 Nucleotide acid and deduced amino acid sequence of *DcBCH1* from ‘Kurodagosun’**

The blue font and the red font indicate the different nucleotides and amino acids between the *DcBCH1* sequence from ‘Kurodagosun’ and ‘Junchuanhong’, respectively.

1 atggcggccggaatttcggcggcctc**a**agctcc**a**cctcattctctctgggccgcaacccatttctcgggcccaaccccatttggctcttc

M A A G I S A A S S S **T** S F S L G R N P F L G P N P I W L F

91 gctcc**t**tccgtccgaaaactaa**g**ccc**a**tct**a**t**c**cggttcaaacaaaagagcttaaccactgtttgttttgtggtggag**c**cccgaaatgat

A P S V R K L **S** P S **I** R F K Q K S L T T V C F V V E **P** R N D

181 agctcgg**t**taagcccgaaaataacgc**g**gaccgagatgaagtgagtcgcgaggagattgaggcggggagtt**c**ttccgtacgagttga**a**gag

S S **V** K P E N N A D R D E V S R E E I E A G S **S** S V R V E E

271 agaagagcgaggaagaagtcggagaggtt**c**acgtatttggtggcggc**g**gttatgtcgagtcttggcattacttctatggctgt**a**ttggcg

R R A R K K S E R F T Y L V A A V M S S L G I T S M A V L A

361 gtttattacagattctcttggcaaatggagggtggcgaaattccgtattcggagatgattggtacattcgctctttctgttggtgctgct

V Y Y R F S W Q M E G G E I P Y S E M I G T F A L S V G A A

451 gtggg**a**atggaattttgggcgagatgggcacatgaagcc**t**tgtggcatgcttcgttgtggcatatgcacgagtcacaccataaacc**a**aga

V G M E F W A R W A H E A L W H A S L W H M H E S H H K P R

541 gaaggagcatttgagctgaatgatgtatttgcaataatgaacgc**a**gttcc**a**gcaatagctttgctagcttatggtttcttccacaaaggc

E G A F E L N D V F A I M N A V P A I A L L A Y G F F H K G

631 tattttccaggtctctgttttggtgcgggcctgggaatcac**g**gtatttggaatcgcttacatgtttgt**a**cacgatggtcttgttcataaa

Y F P G L C F G A G L G I T V F G I A Y M F V H D G L V H K

721 cgatttccggtaggtc**t**cat**t**gc**t**gatgtcccttactt**c**agaaaagttgctgctgctcatcagctgcaccacatggaaaagttcaaagga

R F P V G **L** I A D V P Y F R K V A A A H Q L H H M E K F K G

811 gtcccatatgggttgtttttgggtcctaaggaagttgaggatgtgggaggacacgaagcgttagaattggagatcaacagaagaatcaag

V P Y G L F L G P K E V E D V G G H E A L E L E I N R R I K

901 tcatctgcttccagagctagccgatcatag

S S A S R A S R S *

**Fig. S2 Nucleotide acid and deduced amino acid sequence of *DcBCH1* from ‘Junchuanhong’**

The blue font and the red font indicate the different nucleotides and amino acids between the *DcBCH1* sequence from ‘Kurodagosun’ and ‘Junchuanhong’, respectively.

**
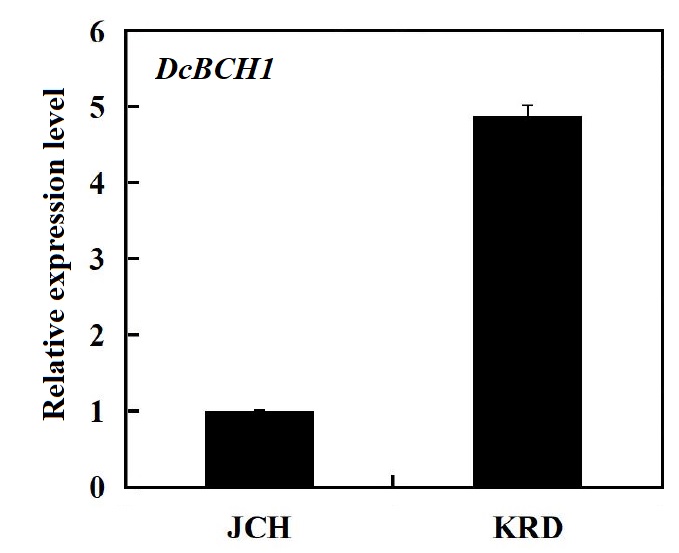
**

**Fig. S3 The expression level of *DcBCH1* in ‘Junchuanhong’ and ‘Kurodagosun’**

JCH, ‘Junchuanhong’; KRD, ‘Kurodagosun’. Bars represent mean standard deviation (SD).
