# Supplementary material for: Overexpression of a carrot BCH gene, DcBCH1, improves tolerance to drought in Arabidopsis thaliana
Source: BMC Plant Biol. 2021 Oct 18;21:475. doi: 10.1186/s12870-021-03236-7 (PMC8522057; doi:10.1186/s12870-021-03236-7)
Supplement: Supplementary file 2 — Additional file 2: Fig. S4 The original gel image for PCR amplification of DcBCH1 from cDNA of non-transgenic (WT) and transgenic plants (OE-3, OE-10, OE-13). [file 12870_2021_3236_MOESM2_ESM.doc]

**Additional file 2:**


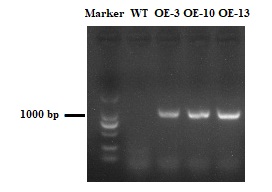


**Fig. S4 The original gel image for PCR amplification of DcBCH1 from cDNA of non-transgenic (WT) and transgenic plants (OE-3, OE-10, OE-13)**
